# Practical issues in treating heavy patients on a LINAC treatment couch

**DOI:** 10.1120/jacmp.v6i1.2077

**Published:** 2005-03-17

**Authors:** Patrick Towns, Blair Free, George Cernica, Matthew B. Podgorsak

**Affiliations:** ^1^ Department of Radiation Medicine Roswell Park Cancer Institute Buffalo New York 14263 U.S.A.

**Keywords:** LINAC treatment couch, weight tolerance

## Abstract

Safe delivery of external beam radiation therapy to patients whose weight approaches the tolerance of a treatment couch presents some challenges, particularly if the couch has been in use for several years and has seen significant wear and tear. An analysis of treatment couch design can identify locations that become stressed and could potentially fail when supporting a heavy patient, leading to serious injury. Some practical methods to decrease the likelihood of couch failure are presented in this work. The design and implementation of a lifting apparatus to support the treatment couch is also described.

PACS numbers: 87.53.Xd

## I. INTRODUCTION

LINAC manufacturers provide tolerances on mechanical components to ensure that their equipment is used within the framework defined by a set of parameters that have been determined to be safe. Likewise, the AAPM Radiation Therapy Committee Task Group 40 Report independently lists tolerances for similar parameters and should be consulted when developing quality assurance procedures for these components.^(1)^ For patient support assemblies, commonly known as treatment couches, there is a limitation on the weight that can be safely lifted and subsequently supported. For the treatment couch in use in our facility (Exact Couch, Varian Medical Systems, Palo Alto, CA), the manufacturer has documented that the weight distributed uniformly over its surface should not exceed 200 kg (440 lbs.).^(2)^ Occasionally, patients with weights approaching this value need to be treated, and while their weight may not exceed the couch tolerance, it may nonetheless be prudent to provide some additional support for the couch during these treatments. This is particularly true if the couch has been in service for a number of years and has seen significant wear and tear on its mechanical parts.

**Figure 1:**
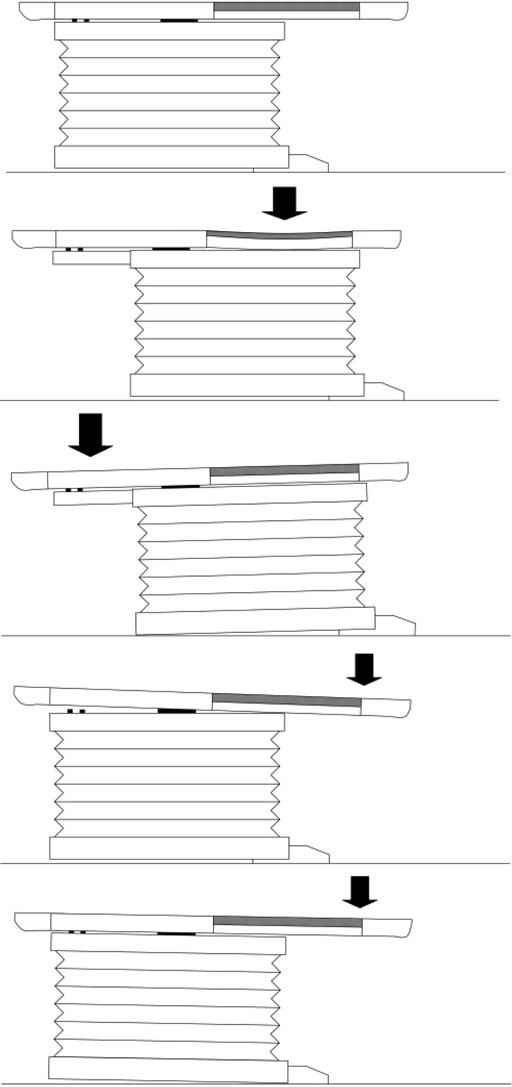
(a) Schematic diagram of a conventional treatment couch showing the tabletop support bracket and the two pairs of connection points between the couch base and tabletop (b) Flexion of the carbon fiber support rails that can occur when a heavy patient lies on the tabletop (c) Torque causing sag at the foot of the couch base caused by a heavy patient sitting on the distal end of the tabletop (d) Catastrophic separation of the tabletop from the couch base caused by a heavy patient lying in treatment position (e) Torque causing uplifting of the foot of the couch base caused by a heavy patient in treatment position

A schematic diagram of a modern LINAC treatment couch is shown in Fig. 1(a). There are three specific components of the treatment couch that become stressed and can subsequently fail when supporting a heavy patient. First, the tabletop and its support rails are made of carbon fiber that can flex due to increased weight bearing and then rub against the couch base (Fig. 1(b). This friction prevents smooth movement of the couch top over the base, making longitudinal and lateral couch top adjustments required for fine patient positioning extremely difficult. We have witnessed tabletop flexing of up to 4 mm when supporting a heavy patient, resulting in very difficult couch movement.

Second, the couch base is usually cantilevered to the frame of the LINAC such that the entire couch assembly floats over the floor. When a heavy patient sits on the end of the couch distal to the gantry, significant torque is applied at the pivot point, as shown in Fig. 1(c). In our experience, we have seen the couch base sag up to 4 mm under these circumstances, exceeding the recommended 2‐mm sag in couch position suggested by Task Group 40.^(1)^ The stresses on the pivot point produced by these sag forces could potentially result in failure at the point where the couch base attaches to the pit bearing.

Finally, with a heavy patient in treatment position, any malfunction at the points where the tabletop attaches to the base or a failure of the tabletop itself, as demonstrated by Fig. 1(d), will result in serious injury to the patient. Furthermore, with the tabletop extended in treatment position, there is significant torque applied at the pivot point where the base attaches to the pit (Fig. 1(e). We have seen the foot of the couch base lift by up to 3 mm relative to its normal position when supporting a heavy patient in treatment position. Similar to the case with a heavy patient sitting at the foot of the couch described above, the stresses produced by an extended tabletop can result in failure at the couch pivot point.

This note describes several simple solutions that, along with the design, development, and implementation of a novel support lift, will help to ensure the safety of heavy patients undergoing external beam radiation therapy.

### II. RESULTS AND DISCUSSION

**Figure 2:**
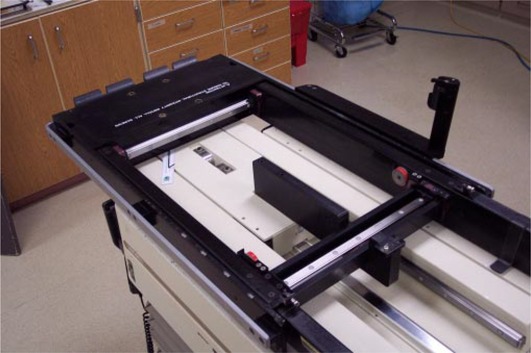
Gap between the tabletop and couch base resulting from placement of the 3‐mm thick shim at the tabletop connection points underneath the block in the center of the figure attached with the two screws to the couch base. The gap is demonstrated by introducing a 2‐mm diameter Allen key between the tabletop and couch base, and compensates for compression of the tabletop support rails.

To properly prepare the treatment couch for treating a heavy patient, it is necessary to separately address each of the structural issues mentioned above. In our practice, tabletop compression and flexing were compensated by adding 3‐mm thick steel shim stock to the two attachment points between the tabletop and the base (Fig. 2). This permanent modification raised the tabletop by 3 mm relative to the couch base, allowing the necessary clearance for a compressed and/or flexed tabletop to move freely over the base. As expected, the couch height parameter had to be recalibrated relative to isocenter to take account of the slight increase in tabletop height.

**Figure 3:**
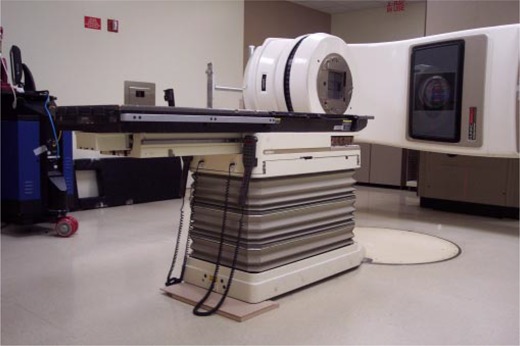
Block of wood placed between the couch base and floor to prevent couch sag with a heavy patient sitting at the foot of the couch

The stress produced at the pivot point of the couch base with a heavy patient sitting near the foot of the tabletop can be alleviated by placing a wooden block of the appropriate thickness between the couch base and the floor. This simple solution, shown in Fig. 3, prevents torque at the location where the couch base attaches to the pit. With the wooden block in place, however, rotating the couch is not possible. If multiple couch angles are required for treatment, then the wooden block can be removed temporarily with the patient in treatment position, since the torque direction at the couch pivot point is reversed with the tabletop in treatment position. The block should be replaced upon completion of treatment to support the couch during unloading of the patient when the torque at the pivot point once again becomes significant.

The third aspect of couch design is arguably the most critical from a patient safety perspective. There are significant stresses present on the couch top when a patient lies uplifted in treatment position with the couch top fully extended (proximal to the gantry). Clearly, some type of external support positioned appropriately under the extended tabletop would be helpful in preventing a catastrophic couch failure that would almost surely result in serious patient injury. There are a few issues to consider when designing such a support. First, the apparatus should interfere minimally with gantry motion and should be very easy to maneuver into position. The support apparatus should not attenuate the beam or produce scatter radiation by impinging on the treatment portal. Finally, the apparatus needs to allow some tabletop movement when deployed in order to permit patient positioning.

**Figure 4:**
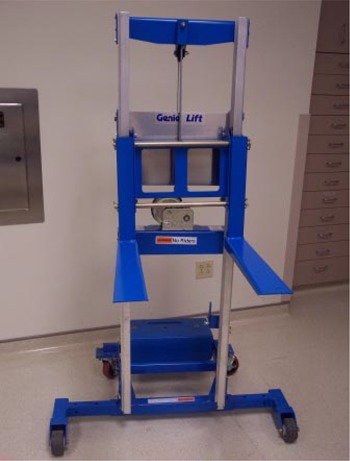
Commercial version of the lift purchased to support the tabletop. The separation between the floor casters is variable to provide a stable base. The forks can also be configured in multiple ways.

**Figure 5:**
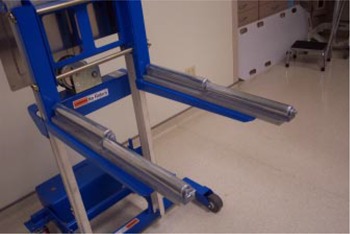
The rollers added to the forks. The rollers are offset to provide maximum support for the couch when supporting a heavy patient. The rollers also allow longitudinal motion even when the lift is supporting the tabletop.

**Figure 6:**
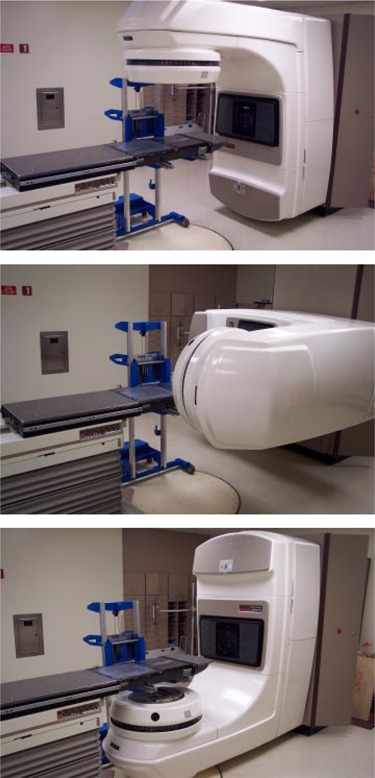
Support lift in position to allow treatment of fields with gantry angles from 0° to 180°. Note that no shifting of the support lift is necessary to treat fields within this range of gantry angles. Shown are fields with gantry angles of (a) 0°, (b) 90°, and (c) 180°.

The approach chosen at our facility was to modify a commercially available lift (GenieLift G‐4, Genie Industries, Redmond, WA) to fit the constraints described above. This unit, shown in Fig. 4, comes with a lifting system with a 227‐kg (500‐lb.) capacity and is comprised of a hand crank with a 2.5‐cm (1‐in.) per turn height adjustment and two forks that can be attached to various points on the lifting mechanism.^(3)^ The lift's stance is very wide, providing stable support, even with the forks attached 45 cm (17.7 in.) apart. Four metal rollers covered with a thin, soft fabric film are mounted on the forks to allow for tabletop longitudinal movement while the lifting mechanism actively supports the couch, as shown in Fig. 5. The rollers are attached using set screws and can be leveled to provide maximum support to the couch rails. If lateral fine‐tuning of patient position is required, the support can be slightly lowered, temporarily releasing the couch top, and then repositioned once the patient is lined up appropriately. When used, the support lift is locked in place close to the couch, and, as mentioned above, substantial gantry rotation is possible before repositioning of the support becomes necessary. In our practice, we have been able to treat all fields within a 180° gantry rotation, enabling the treatment of three of four fields in a standard four‐field box technique without moving the support lift. Figures 6(a) to (c) show the support lift in place with the gantry set at 0°, 90°, and 180°.

## III. SUMMARY

**Figure 7:**
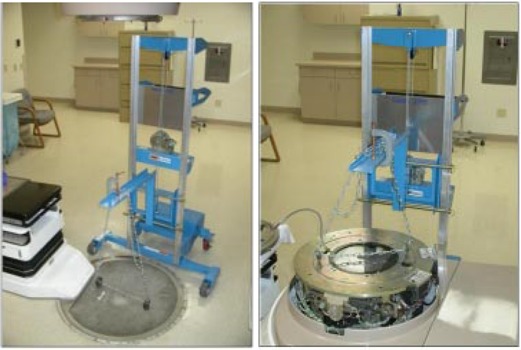
Use of the lift for repair and periodic maintenance of a LINAC. Shown are (a) the lift set up for removal of the pit cover to allow repair work to couch electronics and (b) the lift configured to pull the collimator assembly from the treatment head.

The support lift described above has proven to be invaluable in protecting heavy patients from injury due to couch failure, and has no doubt also minimized couch wear and tear during these treatments. We have made further use of the support lift during routine LINAC maintenance and repair projects that require lifting of heavy items. Figure 7(a) demonstrates the removal of the pit assembly from the floor (the forks were appropriately repositioned). This is a task usually done manually and clearly is much safer when done in conjunction with the support lift. During couch maintenance, the support lift can also be used to raise one end of the couch to place rollers underneath to facilitate movement of the couch away from the pit. Lifting the collimator assembly from a treatment head, as shown in Fig. 7(b), can also be accomplished using the support lift. This task has been done manually, and the risk of personal injury to engineering and physics staff is greatly reduced using the lift.

The capital cost of the support lift is on the order of a thousand dollars, with modifications adding slightly to the cost. In our opinion, the added safety for patients and staff afforded by use of this apparatus is well worth the investment.
